# γ neuromodulations: unraveling biomarkers for neurological and psychiatric disorders

**DOI:** 10.1186/s40779-025-00619-x

**Published:** 2025-06-27

**Authors:** Zhong-Peng Dai, Qiang Wen, Ping Wu, Yan-Ni Zhang, Cai-Lian Fang, Meng-Yuan Dai, Hong-Liang Zhou, Huan Wang, Hao Tang, Si-Qi Zhang, Xiao-Kun Li, Jian-Song Ji, Liu-Xi Chu, Zhou-Guang Wang

**Affiliations:** 1 Affiliated Cixi Hospital, Wenzhou Medical University, Ningbo, 315300 Zhejiang China; 2 State Key Laboratory of Brain and Cognitive Sciences, Laboratory of Neuropsychology & Human Neuroscience, the University of Hong Kong, Hong Kong, 999077 China; 3 The First Affiliated Hospital of Wenzhou Medical University, Wenzhou, 325035 Zhejiang China; 4 Oujiang Laboratory (Zhejiang Lab for Regenerative Medicine, Vision, and Brain Health), National Key Laboratory of Macromolecular Drugs and Large-Scale Preparation, School of Pharmaceutical Sciences, Wenzhou Medical University, Wenzhou, 325035 Zhejiang China; 5 Rehabilitation Medicine Department, Lishui Central Hospital, Lishui, 323000 Zhejiang China; 6 Department of Psychology, the Affiliated Hospital of Jiangnan University, Wuxi, 214062 Jiangsu China; 7 School of Computer Science and Artificial Intelligence, Changzhou University, Changzhou, 213159 Jiangsu China; 8 Department of Psychiatry, the Affiliated Brain Hospital of Nanjing Medical University, Nanjing, 210029 China; 9 Institutes Sciences Cognitives Marc Jeannerod, CNRS UMR 5229, Lyon, France; 10 Zhejiang Key Laboratory of Imaging and Interventional Medicine, Zhejiang Engineering Research Center of Interventional Medicine Engineering and Biotechnology, the Fifth Affiliated Hospital of Wenzhou Medical University, Lishui, 323000 Zhejiang China

**Keywords:** γ oscillations, Neuromodulation, Cross frequency coupling, Deep brain stimulation (DBS), Transcranial magnetic stimulation (TMS), Neurological disorders, Psychiatric disorders

## Abstract

γ neuromodulation has emerged as a promising strategy for addressing neurological and psychiatric disorders, particularly in regulating executive and cognitive functions. This review explores the latest neuromodulation techniques, focusing on the critical role of γ oscillations in various brain disorders. Direct γ neuromodulation induces γ-frequency oscillations to synchronize disrupted brain networks, while indirect methods influence γ oscillations by modulating cortical excitability. We investigate how monitoring dynamic features of γ oscillations allows for detailed evaluations of neuromodulation effectiveness. By targeting γ oscillatory patterns and restoring healthy cross-frequency coupling, interventions may alleviate cognitive and behavioral symptoms linked to disrupted communication. This review examines clinical applications of γ neuromodulations, including enhancing cognitive function through 40 Hz multisensory stimulation in Alzheimer’s disease, improving motor function in Parkinson’s disease, controlling seizures in epilepsy, and modulating emotional dysfunctions in depression. Additionally, these neuromodulation strategies aim to regulate excitatory-inhibitory imbalances and restore γ synchrony across neurological and psychiatric disorders. The review highlights the potential of γ oscillations as biomarkers to boost restorative results in clinical applications of neuromodulation. Future studies might focus on integrating multimodal personalized protocols, artificial intelligence (AI) driven frameworks for neural decoding, and global multicenter collaborations to standardize and scale precision treatments across diverse disorders.

## Background

Neuromodulation has emerged as an essential therapeutic strategy in the treatment of neurological and psychiatric disorders [1–3]. This non-pharmacological treatment strategy is specialized in clinical conditions where traditional pharmacological or behavior modifications have adverse reactions and limited effectiveness, such as in Alzheimer’s disease (AD), Parkinson’s disease (PD), epilepsy, and depression [4–6]. By targeting brain regions and neural circuits, neuromodulation provides the perspective to directly influence abnormal brain states, thereby improving cognitive, emotional, and motor phenotypes in damaged individuals [7–9]. Since the effectiveness of these interventions might rely on their capability to modulate the brain’s neuronal oscillations across different neuronal oscillations, comprehending the relationship between neuromodulation and oscillatory activity is critical for enhancing treatment outcomes.

γ oscillations are progressively identified as vital in recognizing the neural basis underlying psychological and cognitive processes such as perception, attention, memory, and motor functions. These oscillations have the potential as neurobiological markers for early detection, identification, and intervention of various brain disorders, offering the understanding of the dysregulated neural dynamics underlying various clinical symptoms [10–13]. Alterations in γ oscillations are identified to be relevant to adaptions in cognitive processes, emotional regulation, and neural plasticity [14–18]. Imbalances between excitatory and inhibitory processes, as mirrored in raised γ oscillations in the visual cortex including GABAergic interneurons, underscore the relevance of γ rhythms in maintaining neuronal homeostasis [19, 20]. Besides, γ oscillations are modulated by metabotropic glutamate receptors (mGluRs), which also contribute to cognitive functions such as perception and attention [21–24]. As BF GABAergic projection neurons, particularly those containing parvalbumin (PV), play a critical role in triggering cortical γ oscillations. Optogenetic stimulation of BF PV neurons in mice preferentially increases cortical γ band oscillations (GBO) by entraining cortical oscillators at a resonant frequency of approximately 40 Hz, while inhibition of these neurons reduces GBO power [25]. This mechanism is independent of cholinergic neurons and likely involves synchronization of cortical PV interneurons. These findings highlight BF PV neurons as a potential therapeutic target for disorders involving abnormal γ oscillations, such as schizophrenia [25]. Overall, γ oscillations have rich biological implications in coordinating complex brain functions, positioning them as promising targets for neuromodulation [26–29].

Notably, γ oscillations have been linked to a series of neurological disorders, such as neurodegenerative disorders like AD and PD [30]. From the micro-perspective, decreased γ oscillations affecting microglial features have been found to precede plaque development in AD [31]. γ oscillations, generated by striatal cholinergic interneurons, play a crucial role in influencing movement actions within cortical-striatal circuits [32]. γ oscillations of inhibitory nerve cells after stroke have been observed to undermine vascular and lead to behavioral disorder [33]. Besides, γ oscillations have been linked to sensory perception, working memory, and motor control functions in patients with PD, and enhancing the γ oscillations could increase the plasticity of the primary motor cortex in PD [34, 35]. Overall, the relationship between disrupted γ activity and abnormal brain functions of neurological disorders has been identified by neural evidence from macro- and micro-perspectives, emphasizing the further need for precise treatment with γ oscillatory neuromodulation.

Besides, disturbances in γ oscillations have been highly related to a wide variety of psychiatric symptoms, with the restoration of these oscillations emerging as a prospective healing approach [36]. Modified γ activity has been related to psychotic symptoms in drug-naive patients revealing relationships between γ oscillatory patterns and the remaining symptoms [37–40]. Decreased γ oscillations of the limbic cortex have been observed in mice presenting anxiety and anhedonia-like habits [36, 41], implying the relevance of oscillatory disruptions in schizophrenia. Furthermore, γ oscillations could help discriminate different disease states in mental disorders, such as anxiety, depression, or suicide risk [42–44]. Emerging studies indicate that modulating γ activity might alleviate many psychiatric symptoms in both animal models and humans [45–47]. Notably, previous studies highlight the role of basal forebrain (BF) γ oscillations in modulating the default mode network (DMN), a brain network linked to internally focused cognition and dysregulated in brain functional disorders. BF γ oscillations are elevated during quiet wakefulness and suppressed during externally directed tasks, mirroring DMN activity patterns [48, 49]. In rodents, BF γ coherence with the anterior cingulate cortex (ACC), a key DMN hub, suggests functional influence [48]. A study confirmed that BF drives DMN changes during rest-task transitions, while the mediodorsal thalamus engages during internally focused cognition [49]. These findings underscore BF γ modulation as a therapeutic target for DMN-related disorders like epilepsy and depression, offering mechanistic insights into psychiatric symptoms.

Despite the growing understanding of γ oscillations in neurological and psychiatric disorders, there is a significant gap in research specifically addressing the role of γ neuromodulations as a treatment strategy. The present review aims to bridge the gap by systematically examining how γ oscillations affect neurological and psychiatric conditions and the potential benefits of γ neuromodulations for patients. By thoroughly assessing the existing literature on γ neuromodulations from clinical perspectives, and identifying their physiological mechanisms and intervention outcomes, this review seeks to enhance our understanding of γ neuromodulations as promising therapeutic strategies, ultimately improving the efficacy of treatments for neurological and psychiatric diseases.

## Exploring brain dynamics for γ oscillations of the neuromodulations

**Fig. 1:**
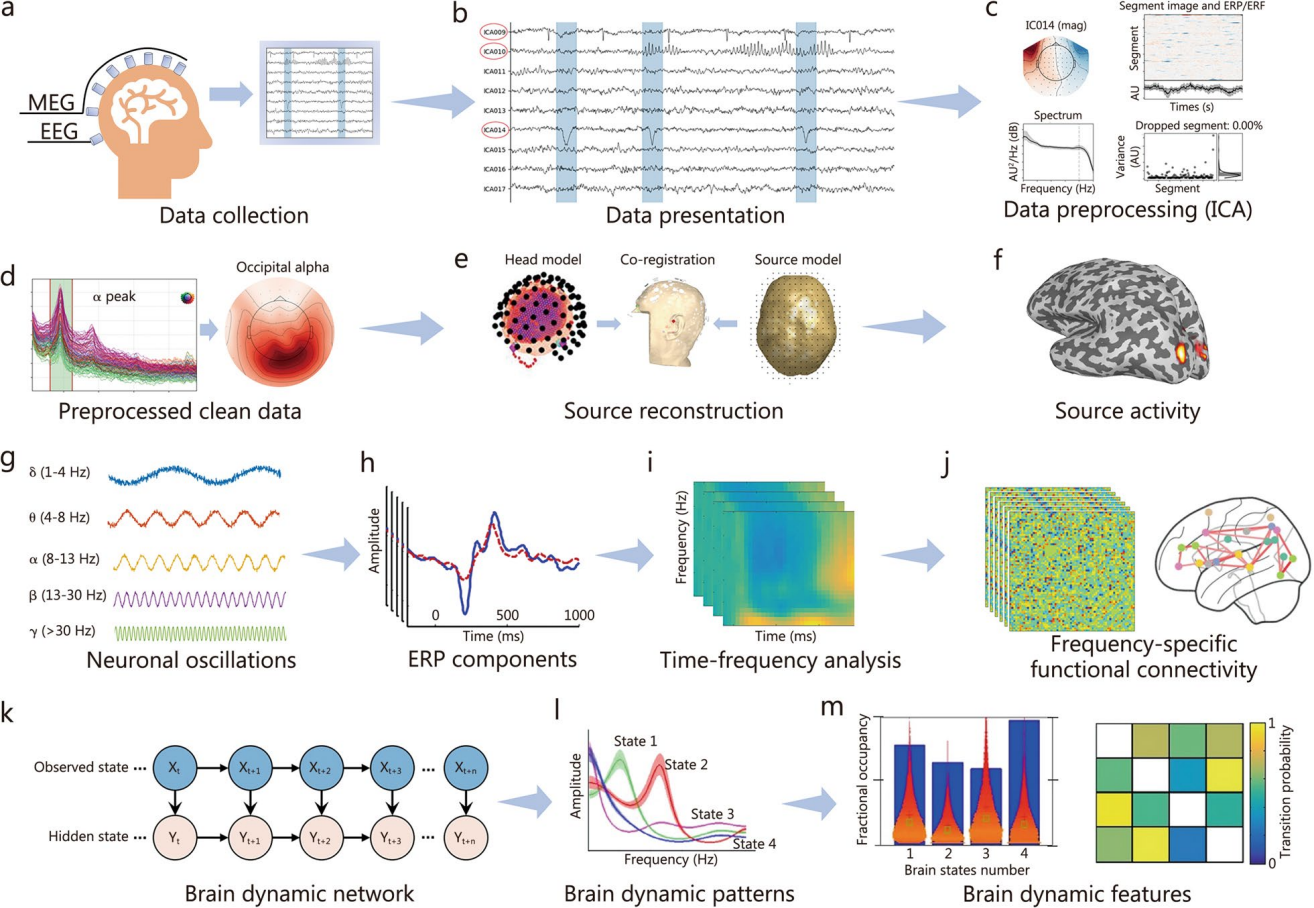
Strategies for collecting and analyzing neuronal oscillations. **a** Data collection procedure using EEG/MEG.** b** Data presentation and reduce artifacts.** c** ICA for extracting clean data. **d** Preprocessed clean data in sensors (visual α oscillations). **e** Source reconstructions.** f** Source power (visual α oscillations). **g** The presentation for neuronal oscillations. **h** ERP analysis. **i** Time–frequency analysis.** j** Frequency-specific functional connectivity, including connectivity matrices and brain connectivity maps. **k** Brain dynamic models (Hidden Markov Model). **l** Brain dynamic patterns describing the power spectra of different brain states. **m** Brain dynamic features including fractional occupancy and transition probability of each brain state. EEG electroencephalogram, MEG magnetoencephalography, ICA independent component analysis, ERP event-related potential

To build an elaborate neuromodulation system of neuronal oscillations, the essential procedures include acquiring, preprocessing, and analyzing the oscillatory activity data (Table 1). In the data acquisition process, various tools are utilized to optimize the collection of neuronal oscillations, including advanced electroencephalogram (EEG) systems equipped with dense electrode arrays and sophisticated Magnetoencephalography (MEG) sensors, which rely on superconducting quantum interference device (SQUID) technology [50–52] (Fig. 1a). After data collection, preprocessing at the channel level is essential to clean the raw data and reduce artifacts like eye blinks muscle activity, and environmental noise [53, 54] (Fig. 1b). This preprocessing pipeline involves techniques such as band-pass filtering, independent component analysis (ICA), and automatic artifact rejection to ensure high-quality data [55, 56] (Fig. 1c, d). Source reconstruction further refines this by constructing head models (based on the individual magnetic resonance imaging data) and source models (to localize active brain regions), enabling the identification of brain areas that display altered oscillatory patterns [57–59] (Fig. 1e). After the source localization, oscillatory activity can be mapped from the sensor space to the brain’s voxel or regional level (Fig. 1f). However, it is important to note that source localization relies on inference under multiple assumptions, and the results may not always fully or accurately reflect actual brain activities.

**Table 1:** Overview of procedures for analyzing γ oscillations

Procedures	Description	Key techniques	Application
Data acquisition	Collection of neuronal oscillation data	Neuroimaging tools, such as Electroencephalogram (EEG), Magnetoencephalography (MEG)	Optimizes data collection
Preprocessing	Cleaning raw data and reducing artifacts	Band-pass filtering, independent component analysis (ICA), artifact rejection	Ensures high-quality data
Source reconstruction	Localizing active brain regions based on MRI data	Beamforming, head model, source model	Identifies oscillatory patterns in brain region levels
Frequency-band analysis	Examining oscillations in specific frequency bands	Event-related potential (ERP), time–frequency analysis	Analyzes frequency-specific brain activities
Functional connectivity analysis	Assessing synchronization between brain regions	Coherence, phase-locked value (PLV)	Reveals network-level communication
Dynamic modelling	Understanding the brain dynamics	Hidden Markov Model (HMM), sliding window	Captures transient brain states

Neuronal oscillations in specific frequency bands, such as θ, α, β, and γ, dynamically change in response to cognitive tasks or external stimuli (Fig. 1g), offering detailed insights into localized brain activity. Common methods for analyzing these oscillations, including event-related potential (ERP) and time–frequency analyses, are particularly useful for examining brain power within specific temporal periods and frequency bands, typically focused on one specific brain region or sensor [60, 61]. ERP analysis measures brain responses that are time-locked to sensory or cognitive events, revealing how oscillatory power in a single region changes in response to stimuli (Fig. 1h). Similarly, time–frequency analysis quantifies how power in specific frequency bands evolves within a given brain region or sensor [62–65] (Fig. 1i). Techniques like wavelet transform and short-time Fourier transforms are used to decompose the signal into its time–frequency components, allowing the tracking of oscillatory power fluctuations [66–68].

To explore relationships between different brain regions, frequency-specific functional connectivity methods, such as coherence and phase-locking value (PLV) [69–71], are commonly applied to assess synchronization at specific frequencies [72–74] (Fig. 1j). These methods offer insights into network-level communication by measuring how different regions of the brain interact and coordinate their oscillatory activity, which is crucial for understanding the brain’s functional architecture and how different areas collaborate during cognitive tasks [75–77]. Beyond connectivity, dynamic approaches such as the Hidden Markov Model (HMM) provide a further understanding of brain networks by modeling brain states as discrete, temporally evolving entities (Fig. 1k) [78–80]. These models enable the identification of transient brain states and their transitions over time, capturing the dynamic nature of brain activity [81–83] (Fig. 1l). Key descriptors from HMM analysis, such as fractional occupancy (the time the brain spends in each state) and state transition matrices (mapping probabilities of transitions between states), provide valuable information about the stability, flexibility, and adaptability of neural networks [84, 85] (Fig. 1m). To accurately extract γ activity from the human brain, utilizing dynamic algorithms to capture comprehensive features is a promising approach. Monitoring changes in dynamic features enables the detailed evaluation of the performance of the neuromodulation system, which can be conducted in both real-time (online) and retrospective (offline) systems.

Combining this dynamic framework with time–frequency power analysis and functional connectivity methods could create a state-of-the-art approach to studying γ oscillations in the neuromodulations [86–88]. This integrated perspective enhances our understanding of stable and transient neuronal activity patterns, revealing how neuromodulation interventions influence cognitive ability and its underlying functional architecture.

## Key neuromodulation strategies in modulating γ oscillations

Neuromodulation technologies regulating neuronal oscillations have obtained significant attention in the clinical neuroscience field [89]. Non-pharmacological treatments such as repetitive transcranial magnetic stimulation (rTMS), transcranial alternating current stimulation (tACS), transcranial direct current stimulation (tDCS), neurofeedback, and deep brain stimulation (DBS) present promising strategies for modulating neuronal oscillations and improving cognitive and emotional functions across various disorders [90–97].

**Fig. 2:**
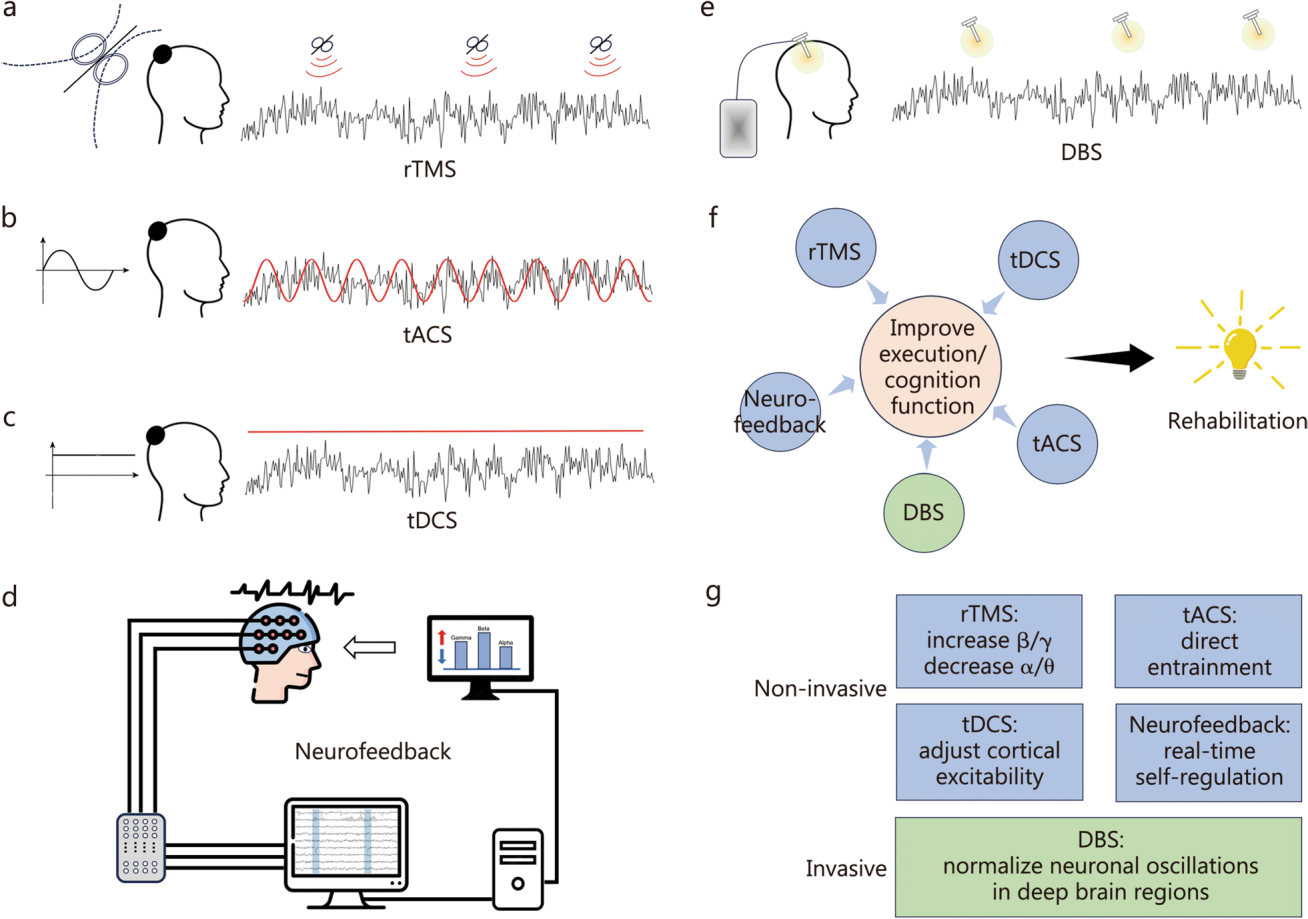
Schematic representation of neuromodulation technologies. Schematic representation of non-invasive neuromodulations rTMS (**a**), tACS (**b**), tDCS (**c**), Neurofeedback (**d**), and invasive neuromodulation DBS (**e**). **f** Neuromodulation treatments enhance cognitive and executive functions, which in turn leads to the rehabilitation of clinical symptoms. **g** Key features for non-invasive and invasive neuromodulation. rTMS repetitive transcranial magnetic stimulation, tACS transcranial alternating current stimulation, tDCS transcranial direct current stimulation, DBS deep brain stimulation

Each of these techniques influences brain activity in unique ways, assisting in the restoration of brain executive or cognitive functions that are often disrupted in both neurological and psychiatric disorders. As illustrated in Table 2, TMS could enhance high-frequency oscillations (γ, β) and simultaneously reduce low-frequency (α, θ, δ) rhythms [98–102] (Fig. 2a). This modulation might adjust the neural communication within frontal-limbic circuits, improving mood stabilization and impulse control [103–105]. Therefore, rTMS has the potential to reduce psychotic symptoms in individuals with treatment-resistant conditions [106].

**Table 2:** Overview of neuromodulation technologies in neurological and psychiatric disorders

Neuromodulation technology	Mechanism of action	Applications	Key features	Limitations
Transcranial magnetic stimulation (TMS)	Non-invasive magnetic pulses to modulate cortical excitability	Depression, obsessive–compulsive disorder, stroke recovery	Precise cortical stimulation, non-invasive	Limited depth penetration, variable individual response
Deep brain stimulation (DBS)	Electrical stimulation via implanted electrodes	Parkinson’s disease, epilepsy, depression	Highly targeted, adjustable stimulation intensity	Invasive, risk of infection, surgical complications
Transcranial direct current stimulation (tDCS)	Low electrical current is applied to the scalp	Depression, anxiety, stroke rehabilitation	Non-invasive, relatively low cost	Limited spatial resolution, inconsistent outcomes
Transcranial alternating current stimulation (tACS)	An alternating current is applied to the scalp to modulate oscillations	Schizophrenia, depression, cognitive enhancement	Targets specific frequency bands (e.g., γ, θ)	Inconsistent effects, poorly understood long-term effects
Neurofeedback (NFB)	Real-time feedback based on brain activity to encourage self-regulation	Attention deficit hyperactivity disorder, anxiety, depression	Allows self-regulation of brain functions through training	Non-invasive, training-based therapy

Through direct entrainment, tACS can synchronize neuronal oscillations with the applied alternating current, offering a targeted way to influence specific frequency bands [107–109] (Fig. 2b). This technique can also modulate γ oscillations [110–112]. By restoring the γ rhythmic activity of disrupted brain networks, tACS may improve the processing of emotional stimuli and alleviate feelings of guilt that contribute to suicide risk [95, 113, 114]. Conversely, tDCS adjusts cortical excitability by delivering low-level electrical currents to the brain, either enhancing or inhibiting activity in targeted regions [115, 116] (Fig. 2c). This modulation can correct abnormal oscillatory patterns and improve emotional regulation. By increasing excitability in areas such as the prefrontal cortex, tDCS may promote better cognitive control and emotional resilience [117–119].

Neurofeedback offers real-time self-regulation neuromodulation mechanisms, allowing individuals with various mental disorders to monitor and adjust their oscillatory activity to achieve the desired brain states [120–122] (Fig. 2d). By training individuals to modulate γ oscillatory rhythms, this approach has shown the potential to enhance emotional stability, reduce stress responses, and improve cognitive flexibility [123–125]. For example, EEG-based neurofeedback can alleviate a series of symptoms including emotional dysregulation, cognitive defections, attention difficulties, and motor control disturbance [126–129]. Previous evidence underscores its psychological relevance: approximately 75% of participants can learn γ modulation, with success rates influenced by baseline traits like fluid intelligence [130]. In preclinical AD and older adults, γ-band neurofeedback achieved 71% accuracy in enhancing neural synchronization, though cognitive gains were limited by high baseline performance [131]. Another meta-analytic evidence further supports the clinical relevance of γ neurofeedback, showing a medium effect size (Cohen’s *d* =  − 0.39) for reduced frontal γ power in schizophrenia patients during working memory tasks [132]. Despite these benefits, neurofeedback faces limitations. Success rates vary significantly due to neurophysiological heterogeneity, sample size constraints, and baseline cognitive performance [133]. Predicting outcomes remains challenging, compounded by the time-intensive and costly nature of training protocols [134]. Nevertheless, this patient-centered approach empowers individuals to actively engage in their treatment, fostering healthier brain activity patterns and offering a promising adjunct to conventional therapies.

DBS, as a more invasive technique, directly stimulates deep brain regions, such as the subgenual cingulate or ventral striatum [135–138] (Fig. 2e). By normalizing neuronal oscillations in deep brain areas, DBS might silence local neural activity to alleviate pathological symptoms, as exemplified in patients with PD [139, 140]. For patients with movement disorders and treatment-resistant psychiatric conditions, DBS offers a powerful means of modulating brain functional networks and alleviating motor symptoms (like tremors, rigidity, and shaking) and psychiatric symptoms (depression, anxiety, and mania).

Among the various neuromodulation techniques, both rTMS and tACS can modulate γ oscillations, though their mechanisms and specificity differ. rTMS enhances γ activity as part of broader network changes involving β and θ rhythms, reflecting its systemic neuromodulatory effects [141, 142]. In contrast, tACS directly entrains γ oscillations through phase-locked sinusoidal currents, achieving sharper spectral precision by synchronizing endogenous neural oscillations to the applied frequency [113, 143]. While rTMS induces broader neurophysiological changes, tACS enables more targeted synchronization, highlighting the distinct therapeutic applications of these modalities [144]. Notably, DBS can affect γ oscillatory activity but is more invasive and typically reserved for severe movement disorders and psychiatric conditions. Neurofeedback offers a novel approach for individuals to learn self-regulation of γ activity, yet it requires active participation and may not provide the same level of direct modulation as tACS. Therefore, tACS stands out as the most effective option for directly regulating γ oscillations on most occasions. Together, these non-pharmacological interventions show considerable promise in enhancing cognitive and emotional functions by directly addressing oscillatory imbalances in the brain, providing essential therapeutic options for both neurological and psychiatric disorders (Fig. 2f, g).

## Treatment via γ neuromodulation

### Restoration of cross-frequency coupling (CFC) using γ neuromodulation

**Fig. 3:**
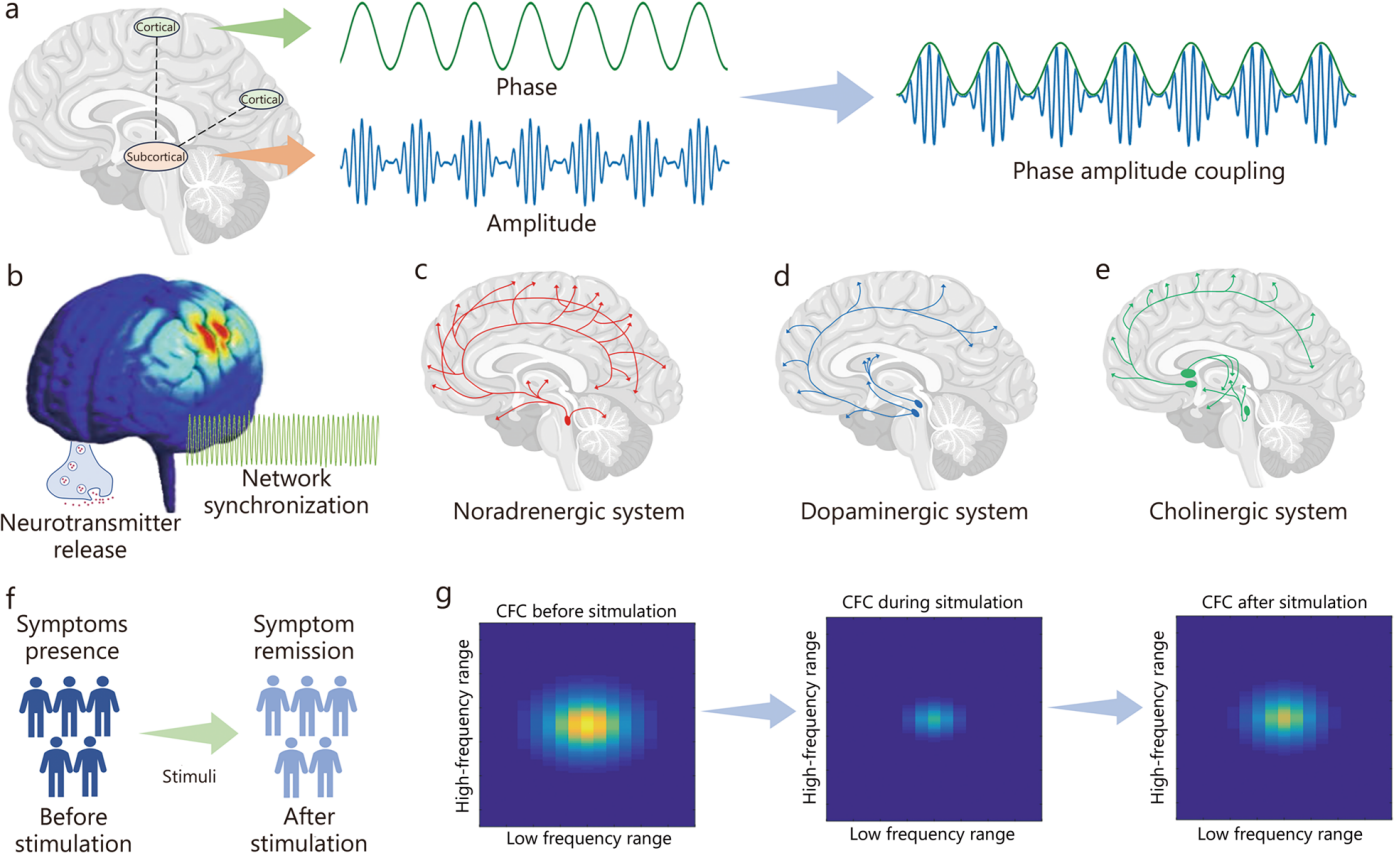
CFC mechanisms and neuromodulation. **a** Phase-amplitude coupling, where the phase of a lower-frequency oscillation modulates the amplitude of a higher-frequency oscillation. **b** Neuromodulation alters neurotransmitter release and brain network synchronization. The noradrenergic system **(c)**, the dopaminergic system **(d)**, and the cholinergic system **(e)** modulate cross-frequency coupling. **f, g** Simulation of phase-amplitude coupling illustrating changes before, during, and after neuromodulation. The neuromodulation intervention effectively restores cross-frequency coupling patterns. CFC cross-frequency coupling

CFC refers to the interaction between neuronal oscillations of different frequency bands, wherein the phase, amplitude, or power of one oscillatory rhythm influences the dynamics of the other [145, 146]. The most widely studied form of CFC is phase-amplitude coupling (Fig. 3a), where the phase of a lower-frequency oscillation modulates the amplitude of a higher-frequency oscillation [147, 148]. θ − γ phase amplitude coupling (PAC) and θ − γ PAC have been extensively examined as critical mechanisms for organizing neural activity across various brain regions [149, 150].

γ neuromodulation can alter the neuromodulatory system by modifying the interactions between different brain oscillations. This impacts neurotransmitter release, synaptic plasticity, and brain network synchronization (Fig. 3b), which are all essential for the regulation of cognition and behavior in neurological and psychiatric disorders. Specifically, major neuromodulatory systems, including the noradrenergic, dopaminergic, and cholinergic systems, exert significant influence on CFC patterns. The noradrenergic system, originating from the locus coeruleus, enhances γ power and modulates CFC during arousal and attentional processes [151, 152] (Fig. 3c). The dopaminergic system, particularly in the ventral tegmental area, plays a critical role in working memory, influencing β − γ coupling in these cognitive functions [153–155] (Fig. 3d). The cholinergic system, via the basal forebrain, modulates θ − γ coupling, essential for memory encoding and retrieval [156–158] (Fig. 3e). Altering CFC through neuromodulatory interventions like TMS and DBS has shown promise in correcting abnormal neural communication [159–162].

CFC has gained considerable attention for its role in understanding the neural mechanisms underlying neurological and psychiatric disorders. In the context of neurological disorders, CFC alterations have been identified in diseases such as PD and AD, where they are associated with motor and cognitive deficits [163–167]. Specifically, disrupted θ − γ PAC is observed in AD [168], while changes in β − γ CFC are linked to motor dysfunction in PD [169]. Besides, CFC has been linked to key neural processes like corticostriatal function and higher-order cognitive activities, both of which are implicated in psychiatric disorders [170, 171]. Such findings point to the broader applicability of CFC as a biomarker across a wide range of psychiatric conditions, with implications for both diagnosis and treatment. Since the application of CFC in neuromodulation holds significant therapeutic potential (Fig. 3f), by targeting specific oscillatory patterns and restoring healthy CFC, these interventions may help alleviate cognitive and behavioral symptoms associated with disrupted cross-frequency communication (Fig. 3g). Specifically, patients may exhibit an over-activated CFC pattern before γ neuromodulation, followed by a rapid transition to an inhibited-activated CFC pattern during stimulation. After the stimulation, the previously over-activated patterns may partially return but remain significantly weakened compared to their initial state, correlating with a reduction in the symptoms of the disorder.

As the understanding of CFC deepens, its role as a diagnostic and therapeutic tool in clinical practice is likely to expand, offering new avenues for personalized and targeted treatments. The integration of CFC-based neuromodulation with emerging evidence on its causal role in cognition, language, and decision-making will pave the way for innovative therapeutic strategies in psychiatric disorders.

### Brain disorders

#### Multisensory 40 Hz γ neuromodulation associated cognitive function in AD

**Fig. 4:**
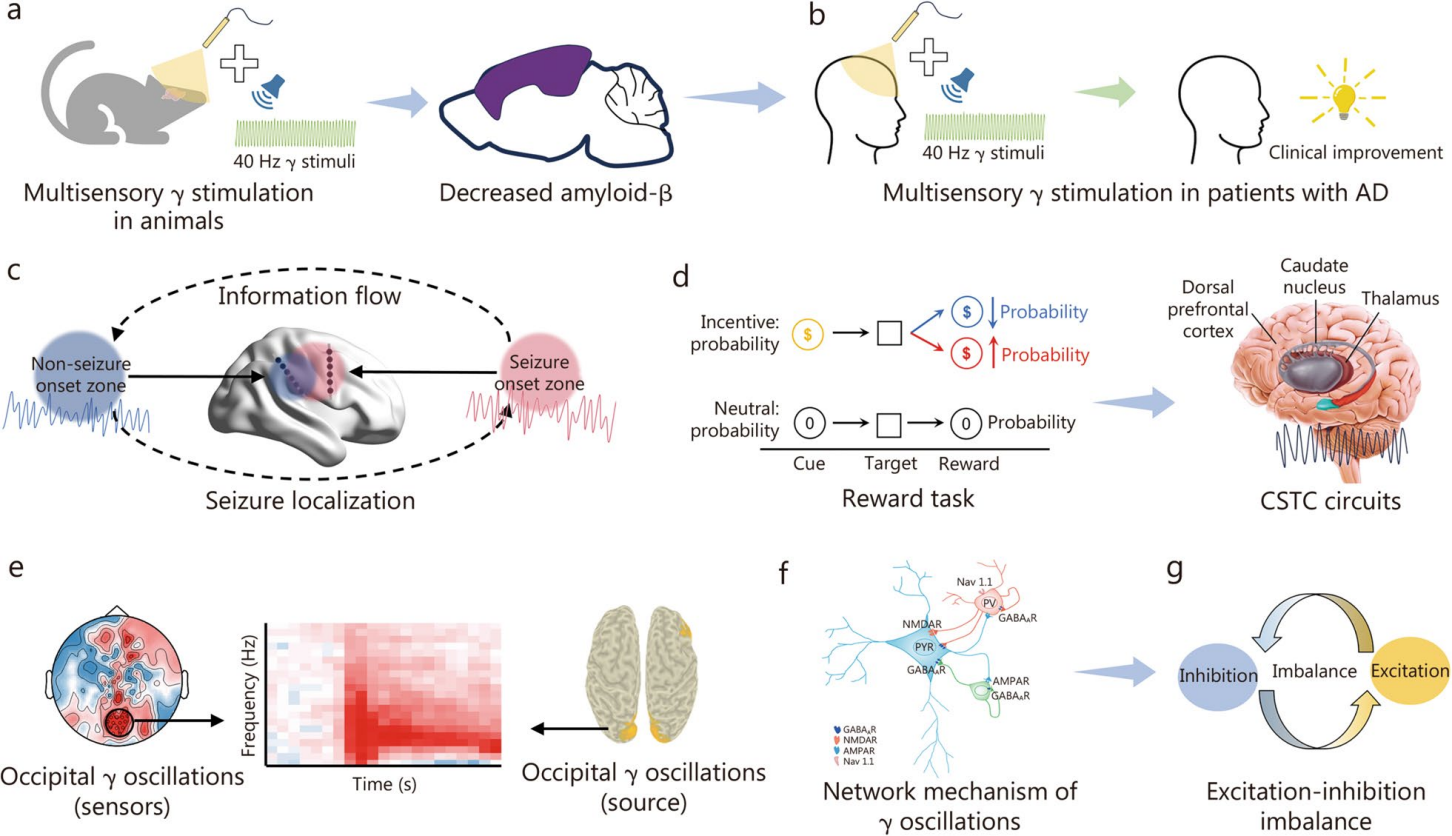
Diverse impacts of γ modulations in neurological and psychiatric disorders. **a** The 40 Hz multisensory stimulation contributing to the clearance of amyloid-β in Alzheimer’s disease mouse models. **b** The 40 Hz multisensory stimulation showed improvements in diverse clinical symptoms in patients with Alzheimer’s disease. **c** Localization of seizure onset zones and prediction of seizure outcomes in epilepsy with γ oscillations. **d** The cortico-striatal-thalamic-cortical (CSTC) circuit shows the role of γ oscillations in obsessive–compulsive disorder during reward-based tasks.** e** Increased γ oscillations of occipital regions in patients with depression and schizophrenia. **f** The biological network mechanisms underlying γ oscillations. **g** Depiction of excitatory-inhibitory imbalance. AD Alzheimer’s disease, CSTC cortico-striatal-thalamic-cortical, PYR pyramidal neurons, PV parvalbumin, GABA γ-aminobutyric acid

γ neuromodulation has emerged as a promising therapeutic approach for cognitive decline in AD. In animal experiments, optogenetic neuromodulation targeting γ oscillations has been explored as a strategy to delay or mitigate cognitive decline in AD patients [172]. Specifically, multisensory γ stimulation at 40 Hz has shown significant potential in reducing AD pathology by decreasing amyloid-β accumulation. This is achieved through suppression of its production, enhanced microglial clearance, promotion of cerebrospinal fluid movement via aquaporin-4 polarization, and increased arterial pulsation and lymphatic vessel expansion, all contributing to amyloid-β clearance [31, 173, 174] (Fig. 4a). These findings highlight the therapeutic potential of 40 Hz γ stimulation as a non-invasive intervention for AD.

From the clinical perspective, early research using DBS applied to the fornix demonstrated cognitive and behavioral improvements in AD patients by activating key regions like the hippocampus and medial prefrontal areas [175]. Non-invasive methods, such as γ rhythmic sensory stimulation (RSS) and tACS, have also shown promising outcomes. Specifically, γ RSS improved memory and reduced brain volume loss in early AD patients [176], while tACS targeting frontal areas enhanced cognitive functions like working memory and cognitive control [95, 177]. tACS has gained popularity due to its cost-effectiveness and portability, though TMS also demonstrated positive effects on γ activity and cognition [178, 179]. New approaches like multisensory stimulation with neurofeedback, which enhance γ activity through light, auditory stimulations, and operant conditioning, have been linked to improvements in working memory and cognitive functions in AD and mild cognitive impairment patients [180–182] (Fig. 4b). γ-band synchronization can also be self-modulated through EEG-neurofeedback, showing potential in the elderly population [131]. Preliminary trials indicate that γ-neurofeedback significantly boosts frontal γ responses, reinforcing its promise as a therapeutic tool for AD-related cognitive decline [183].

#### γ oscillations neuromodulation improving motor function in PD

γ neuromodulation has shown potential as a therapeutic approach for improving motor functions in PD with control-theoretical principles to modulate hippocampal γ oscillations through posterior cingulate cortex stimulation [184]. The effect of stimulation intensity on γ band entrainment was explored, comparing the entrained signal during rest and repetitive movement [185]. Sub-harmonic entrainment of cortical γ oscillations to DBS in PD patients has been recorded via EEG, with γ entrainment proposed as a new biomarker for motor improvement in PD [185, 186].

Additionally, phase-dependent neuromodulation has been investigated, with β-phase and γ amplitude implicated in PD pathology [187]. Fast θ – γ oscillation entrainment in PD patients has shown benefits, especially in the absence of dyskinesia [186]. While DBS is a widely used therapy, its lack of adaptability to changing clinical and neural conditions has been noted [188]. γ power suppression has been linked to tremor reduction in patients undergoing DBS, and cortical γ oscillations have been associated with levodopa-induced dyskinesia in PD [189, 190]. Collectively, these findings advocate for the continued exploration and refinement of γ neuromodulation techniques to enhance motor function and improve the quality of life for individuals with PD.

#### γ neuromodulations in seizure control for epilepsy

In epilepsy, key oscillatory patterns include the spike-wave complex (SWC) and high-frequency oscillations (HFOs) [191]. The previous one is an EEG marker of paroxysmal depolarization shifts, particularly in the absence of seizures, disrupting cognitive functions. The HFOs, classified as ripples(80 – 250 Hz) and fast ripples(250 – 600 Hz), serve as biomarkers for the epileptogenic zone and often occur during slow-wave sleep [192]. The presence of these specific γ components can predict favorable surgical outcomes, emphasizing their importance in identifying cortical areas for effective seizure control [192]. Consequently, γ neuromodulation might be considered a promising therapeutic strategy for epilepsy, facilitating targeted interventions and restoring balance in seizure management. Specifically, it aids in localizing seizure onset zones (SOZ) and predicting seizure outcomes using directional CFC data collected by brief resting-state stereo-electroencephalography (SEEG) data, achieving 88% SOZ localization accuracy and 92% prediction accuracy by analyzing directional information flow between SOZ and non-SOZ in the γ frequency band, offering a faster pathway to effective seizure control [193] (Fig. 4c).

Additionally, dynamic mode decomposition has been used to identify epileptic networks in focal epilepsy patients, highlighting its potential for neuromodulation therapies [194]. Closed-loop brain stimulation studies emphasize γ frequency modulation in non-lesional refractory motor epilepsy, further exploring the neurophysiological effects [195]. γ Knife Surgery has demonstrated non-destructive neuromodulatory effects, backed by both clinical and experimental data, and has been highlighted as a potential treatment option [196, 197]. Other neuromodulation techniques, like DBS and vagus nerve stimulation (VNS), have also shown promise in epilepsy treatment, with VNS desynchronizing epileptiform activity in the γ band and thalamic DBS modulating γ and high-γ oscillations in the hippocampus [198, 199].

Besides, thalamic neuromodulation, particularly DBS targeting the anterior nucleus of the thalamus (ANT), represents an innovative and FDA-approved treatment approach for drug-resistant epilepsy [200]. Bilateral ANT stimulation has been shown to reduce seizures by 40.5% at 3 months and by 75.0% at 5 years, with efficacy best documented for focal onset seizures in the temporal or frontal lobes [201]. Other thalamic sub-nuclei, such as the centromedian nucleus and pulvinar, have also been explored for their potential in treating generalized or multifocal seizures and posterior limbic seizures, respectively [201]. While thalamic DBS is associated with side effects such as paresthesias, mood changes, and memory disturbances, it offers a valuable option for patients with refractory epilepsy who are not candidates for resective surgery [201].

While some studies suggest that low-frequency rTMS and tDCS can impact epileptiform activity, their effectiveness in reducing clinical seizures remains uncertain [202–204]. The variability in results from randomized controlled trials highlights the importance of careful interpretation, as the effects of active rTMS have varied from no benefit to significant improvement [205]. In contrast, invasive techniques such as VNS and DBS show a more definitive pathway for seizure control, with evidence indicating that these methods effectively intervene in comparable γ-related neural pathways. These findings collectively underscore the therapeutic potential of neuromodulation for γ activity in epilepsy management.

### Psychiatric disorders

#### γ neuromodulations within CSTC in obsessive compulsive disorder (OCD)

Neuromodulation has emerged as a helpful approach for alleviating symptoms of OCD, particularly through high-frequency modulation, including β – γ modulation, which has been shown to produce significant and lasting improvements in obsessive–compulsive behaviors [206]. This effect may be linked to maladaptive habit learning, which is a key feature of OCD pathology [207–209]. This finding indicates that by disrupting these entrenched patterns, γ neuromodulation can facilitate a more adaptive response to triggers, thereby reducing the frequency and intensity of compulsive behaviors. Invasive techniques like thermos capsulotomy and γ knife capsulotomy have also been explored, yielding varying outcomes in the OCD treatment [210]. Responsive DBS targeting ventral striatal activity has demonstrated efficacy in reducing OCD symptoms, as have other neuromodulatory approaches, such as γ knife ventral capsulotomy [211–213]. Non-invasive methods, such as TMS, remain experimental but are being investigated as potential treatments for OCD [214].

The cortico-striato-thalamo-cortical (CSTC) circuitry, which is involved in the regulation of habits and goal-directed behavior, plays a central role in OCD, with dysfunction in this loop contributing to the disorder’s repetitive behaviors [215–217]. Modulating this circuit, particularly through pathways involving γ-aminobutyric acid (GABA) between the dorsal medial pallidum and thalamus, has shown promise in improving symptoms [218]. The involvement of reward-related circuits in the CSTC loop suggests that altering the activity in these regions could help in managing the compulsive and repetitive behaviors of OCD (Fig. 4d). Considering the convincing connection between the CSTC circuitry, obsessive–compulsive behavior, and GABA, non-invasive neuromodulation that targets γ oscillations within the CSTC represents a promising therapeutic approach for future OCD treatments.

#### Auditory-driven γ oscillations neuromodulation in schizophrenia

Schizophrenia is a psychiatric disorder marked by significant deficits in working memory and disrupted γ neuromodulation [132]. A novel therapeutic approach to address these deficits is γ Entrainment Using Sensory Stimulation (GENUS), designed to restore impaired neural activity through sensory-driven entrainment. This strategy aims to enhance γ oscillations, thereby addressing cognitive deficits and providing deeper insights into the pathophysiology of schizophrenia [219]. Consistent deficits in γ-range auditory steady-state responses (ASSRs) have also been observed in schizophrenia, signaling underlying preclinical neural impairments [220, 221]. Furthermore, alterations in schizophrenia-related genes have been associated with abnormal γ frequency responses to auditory stimulation [222]. Altered expression of genes involved in GABAergic transmission has been found in schizophrenia, affecting GABA neuromodulation [223]. These findings underscore the intricate interplay between genetic factors and γ oscillatory activity in schizophrenia, highlighting the importance of developing targeted interventions to address γ dysregulation.

TACS has emerged as an effective intervention, given that schizophrenia patients exhibit reduced task-related γ oscillations and diminished synchronization [224]. γ neuromodulation has also been explored through neurofeedback training, showing potential in enhancing working memory in individuals with schizophrenia [225]. Together, these findings emphasize the critical role of γ dysfunction in schizophrenia’s cognitive impairments, suggesting that sensory-driven especially auditory-driven γ modulation using TACS could represent a potential target for the precise therapeutic interventions of schizophrenia.

#### Multimodal γ neuromodulation in depression

γ neuromodulation has emerged as a promising therapeutic approach for depression, showing potential to alleviate both depressive symptoms and cognitive impairments. Enhancing or silencing cortical γ oscillations through olfactory bulb-driven closed-loop neuromodulation has been found to modulate depressive symptoms effectively [226]. γ oscillations, primarily governed by GABA, also play a significant role in the longitudinal changes within depressive circuitry in response to neuromodulation therapies [227]. Additionally, γ neuromodulation has been associated with sustained improvements in working memory and depression symptoms, particularly in patients with stronger expertise-dependent cognitive structures [228]. γ activity, including resting-state EEG γ power and θ – γ coupling, has shown a strong correlation with depression and may be modifiable through interventions such as rTMS [142]. VNS, which is FDA-approved for epilepsy and depression, has also emerged as a promising neuromodulation therapy [229]. Similarly, a recent study has revealed that high-frequency DBS of the habenula has shown potential clinical efficacy in alleviating symptoms of treatment-resistant depression [230].

Recent studies highlight the potential of γ-band modulation in improving treatment outcomes. Dysregulated γ-band functional connectivity induced by the emotional stimuli, particularly within the fronto-parietal control network, may help explain the mechanisms behind neuromodulation therapies. Abnormal γ dynamics, such as those seen in low-γ brain states, have been linked to varying responses to depression treatments [26, 231]. Notably, non-phase-locked γ oscillations (50–70 Hz) of the occipital cortices, which are induced by emotional stimuli, have emerged as potential biomarkers for predicting treatment efficacy in patients with mood disorders [232] (Fig. 4e). Besides, as DMN dysregulation is linked to depressive pathology, emerging evidence indicates that the BF modulates maladaptive self-referential processing through γ-mediated state transitions between rest and task engagement [49]. Targeting BF nuclei (e.g., cholinergic circuits) to normalize γ synchrony could restore emotional balance, consistent with findings that γ dysregulation contributes to disordered cognition across psychiatric disorders, including schizophrenia [25]. These mechanisms offer therapeutic pathways for neuromodulation in psychiatric conditions [48, 233]. These findings underscore the therapeutic promise of γ neuromodulation and its potential for enhancing future treatment strategies in depression and related disorders, with ongoing research needed to fully harness its benefits in clinical practice.

### Neuromodulation of γ oscillations regulating the excitatory-inhibitory imbalance

γ oscillations play a critical role in cognitive and emotional processes, making them valuable biomarkers in both neurological and psychiatric diseases. These oscillations are closely linked to the excitatory-inhibitory (E/I) balance, which is crucial for proper information processing and neural plasticity [234]. γ oscillations are generated through mechanisms like pyramidal-interneuron network γ (PING) and interneuron network γ (ING), where excitatory pyramidal cells and inhibitory interneurons, such as parvalbumin-expressing (PV^+^), somatostatin-expressing (SST^+^), and vasoactive intestinal peptide (VIP) interneurons, interact to produce γ-frequency oscillations [235–239] (Fig. 4f). Disruptions in these networks, such as excessive excitation or impaired inhibition, lead to aberrant γ oscillations, which can reflect maladaptive neural dynamics in neurological and psychiatric diseases [240, 241].

The mechanisms underlying γ modulation involve the interaction of GABAergic (inhibitory) and glutamatergic (excitatory) neurotransmission. GABAergic neurotransmission is particularly important for maintaining γ rhythms, as GABAA receptor activity regulates inhibitory postsynaptic currents (IPSCs) [234, 239, 242, 243]. Conversely, glutamatergic transmission is essential for fast synaptic activity and contributes to γ band generation [244]. Disruptions in either GABAergic or glutamatergic systems can cause uncommon γ oscillations, which are commonly observed in neurological and psychological diseases. For instance, decreases in γ-band ASSRs in schizophrenia are connected to deficits in GABAergic transmission, highlighting the significance of γ modulation for cognitive and psychological processing in these clients [245].

γ oscillations reflect the brain’s capability to maintain an optimum E/I equilibrium, which is vital for both neurological and psychiatric health. Outside stimulations, intrinsic factors like GABA resting degrees, and the structural buildings of the cortex influence this equilibrium [246, 247]. In condition states, the imbalance of excitation and inhibition causes modified γ patterns, adding to cognitive deficiencies and psychological dysregulation [248]. Neuromodulation therapies aimed at restoring γ oscillations offer a promising avenue for treating neurological and psychiatric disorders by addressing these underlying imbalances [249–251]. These therapies, including DBS, TMS, and sensory-based interventions, show the potential to improve clinical symptoms and enhance cognitive functions in neurological and psychiatric diseases. By targeting the disruptions in γ activity, neuromodulation may lead to more effective treatments for a range of physical and psychological health.

### Restoration of γ synchrony via neuromodulation

γ oscillations play a significant role in various neurological disorders, as they are crucial for maintaining neuronal synchrony, which is essential for normal cognitive and motor functions. Neuronal synchrony refers to the coordinated timing of neural firing across different brain regions, enabling effective communication and integration of information essential for complex cognitive tasks and motor control. When synchrony is disrupted, it can lead to impaired functioning and contribute to the pathophysiology of various neurological disorders.

In AD, γ oscillations have been linked to amyloid-β clearance and neuroinflammation [252]. Animal studies have shown that 40 Hz γ entrainment using sensory stimulation, such as light flicker or sound, can reduce amyloid plaques and tau pathology in mouse models of AD, suggesting that γ modulation might slow the progression of neurodegeneration by promoting neuronal synchrony and clearing toxic protein accumulation [31, 173, 174]. By enhancing neuronal synchrony, these interventions may improve the brain’s ability to communicate effectively and manage metabolic waste, which is critical for preserving cognitive functions.

Similarly, in PD, altered γ synchronization is associated with motor symptoms [253]. DBS, particularly in the subthalamic nucleus (STN), helps modulate γ activity, restoring more physiological oscillatory patterns and improving motor function by reducing pathological synchrony within basal ganglia-cortical circuits [254, 255]. In epilepsy, γ oscillations are disrupted by an E/I imbalance, contributing to the hyperexcitable neural activity that leads to seizures [256, 257]. Neuromodulation techniques, such as TMS and DBS, aim to restore γ oscillations by rebalancing excitatory and inhibitory transmission [191]. These interventions stabilize neuronal networks by modulating γ rhythms, effectively re-establishing synchrony among neural circuits and reducing both the frequency and intensity of symptoms. By restoring neuronal synchrony, these therapies help prevent seizure initiation and promote overall neural stability, underscoring the critical role of γ oscillations in maintaining healthy brain function.

In psychiatric disorders like depression and schizophrenia, abnormal γ oscillations are associated with E/I imbalance, which affects emotional regulation and cognitive functions [258–263] (Fig. 4g). Disrupted γ power has been observed in patients with depression or schizophrenia, serving as a potential marker of synaptic homeostasis dysregulation [27, 221, 264–266]. Neuromodulation therapies, such as tACS and DBS, target γ oscillations to restore this balance and alleviate symptoms. By modulating γ activity, these interventions may improve outcomes by enhancing emotional processing and reducing depressive symptoms. The common mechanisms underlying these approaches include the restoration of synaptic plasticity and the facilitation of better neural communication, which together highlight the therapeutic potential of γ neuromodulation across both neurological and psychiatric disorders. This suggests that further exploration of γ oscillation modulation could be key to developing effective treatments for a range of conditions marked by neural dysregulation.

## Limitations

**Fig. 5:**
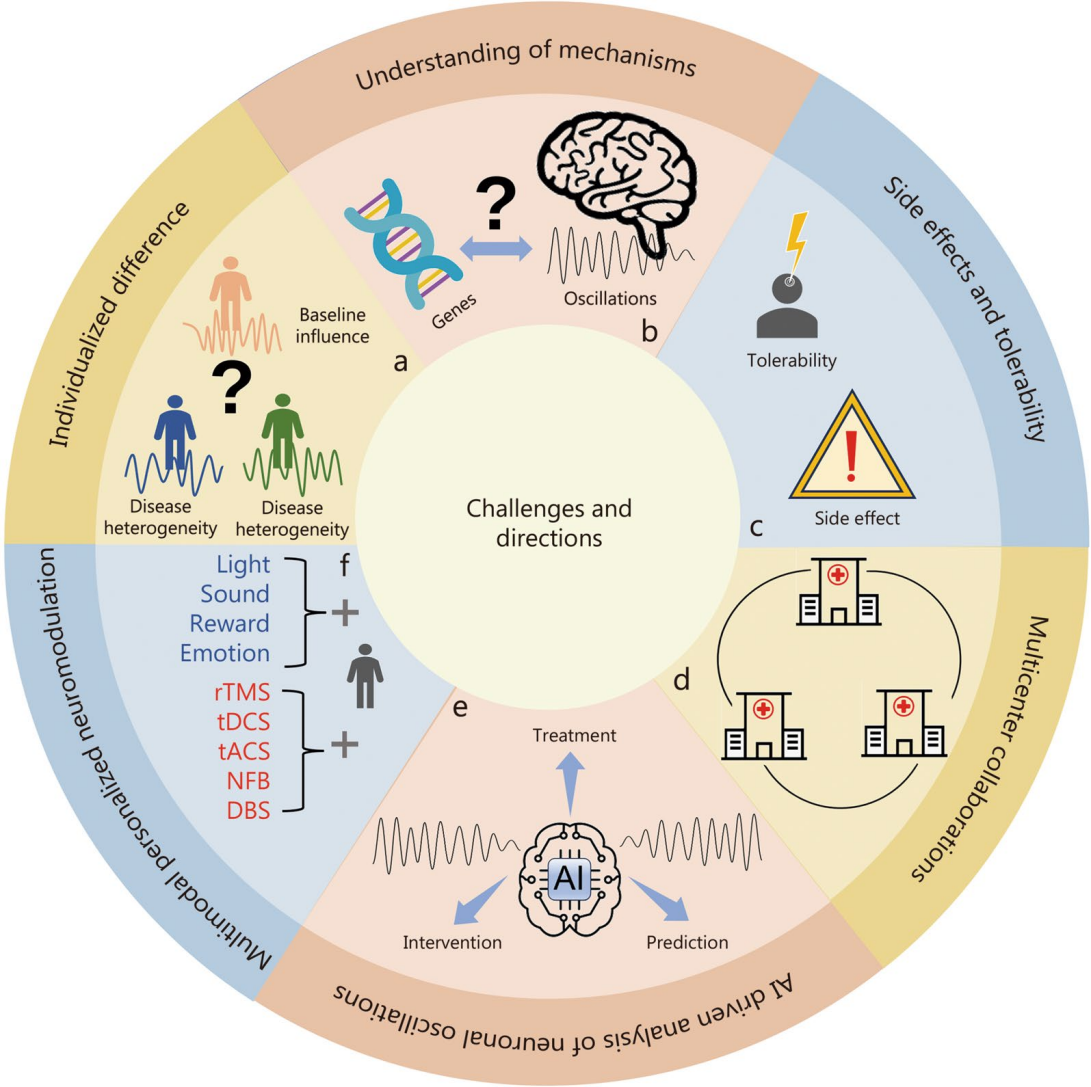
Limitations and future directions in utilizing γ neuromodulations. **a** Individualized differences for γ neuromodulations. **b** Understanding of the neurobiological mechanisms. **c** Side effects and tolerability. Future directions: **d** Multicenter collaborations to enhance treatment efficiency. **e** AI-driven analysis for customized interventions.** f** multimodal individualized methods. rTMS repetitive transcranial magnetic stimulation, tDCS transcranial direct current stimulation, tACS transcranial alternating current stimulation, NFB Neurofeedback, DBS Deep brain stimulation, AI Artificial intelligence

Regardless of the encouraging possibility of γ neuromodulation in neurological and psychological diseases, several limitations remain (Fig. 5a–c). Firstly, individualized differences in response to γ oscillation modulation provide a substantial challenge. The efficacy of therapy can differ substantially across individuals because of variables such as genetic proneness, baseline neural activity, and the intricacy of neurocircuitry involved in each disorder. Tailoring treatments to individual profiles remains difficult, limiting the current capacity for precision therapy. Secondly, there is a limited understanding of the underlying biological mechanisms by which γ oscillations affect cognitive and emotional regulation. While γ oscillations are known to be involved in excitatory-inhibitory balance, synaptic plasticity, and network communication, the specific pathways and interactions that modulate these processes during neuromodulation are not fully understood. This hampers the ability to optimize therapeutic protocols. Technologies such as spatiotemporal multi-omics could help explore the molecular mechanisms underlying these oscillatory disruptions, shedding light on gene expression, epigenetic regulation, and metabolic pathways [267–269]. Lastly, side effects and tolerability pose concerns. γ neuromodulation, whether through rTMS, DBS, or other tools, can cause unintended side effects such as headaches, mood shifts, or cognitive disturbances, with individual tolerability varying widely. Ensuring both safety and efficacy across diverse patient populations remains a critical area for improvement. Addressing these limitations will be key to advancing the clinical utility of γ neuromodulation.

## Future directions

Several promising avenues emerge for subsequent exploration (Fig. 5d–f). Firstly, multimodal personalized neuromodulations are critical. Integrating data from multiple neuroimaging techniques and combining different neuromodulation methods (e.g., rTMS, DBS, tACS) during the multisensory environment (light, sound, emotion, and reward stimuli) could allow for more individualized treatment plans, tailored to the unique neurobiological profiles of patients. This could improve precision and effectiveness by targeting specific neural circuits with optimized parameters [270, 271]. Secondly, artificial intelligence (AI) driven analysis of neuronal oscillations shows significant promise, with established machine learning frameworks identifying neural oscillatory biomarkers for disorders such as post-traumatic stress disorder (PTSD), ASD, and depression [272, 273]. Besides, Artificial neural networks (ANNs) further highlight low-γ oscillations as optimal for decoding behavioral states [274]. The latest advancements utilize large language models (LLMs) to expand the scope of neuronal oscillatory activities. Specifically, large language model meta AI-2 (LLaMa-2) enables semantic reconstruction of visual stimuli from γ-modulated EEG [275], while Generative pre-trained transformer-4 (GPT-4) detects schizophrenia via interpretable EEG analysis, revealing γ-related neural signatures [276]. By integrating neural oscillation data with clinical and cognitive metrics, AI holds the potential to enhance the efficiency of treatment predictions and optimize intervention strategies. Lastly, fostering multicenter collaborations will be essential for advancing the field. Large-scale, cross-institutional studies can provide robust data on the variability of neuromodulation responses across diverse populations. This would help with the growth of standardized procedures and boost the generalizability of findings. Joint networks might also increase the discovery of biomarkers for treatment reactions, consequently refining γ modulation approaches and increasing their restorative reach throughout various neurological and psychiatric conditions.

## Conclusions

In summary, this review highlights the vital role of γ neuromodulation in sharpening executive functions and cognitive processes throughout a range of neurological and psychiatric disorders. By describing the elaborate systems of γ oscillations and their possibility as biomarkers for therapeutic efficacy, we highlight the significance of these characteristics for reliable medical applications. The combination of cutting-edge neuroimaging techniques with targeted neuromodulation strategies offers promising avenues for future research, specifically in refining treatment protocols to enhance patient outcomes. Further investigation into the biological mechanisms driving individual variability in responses to γ modulation will be essential for advancing precision medicine in neuromodulation therapies.

## Data Availability

No new data was applied for the research described in the review.

## Abbreviations

ACC: Anterior cingulate cortex
AD: Alzheimer’s disease
AI: Artificial intelligence
ANNs: Artificial neural networks
ANT: Anterior nucleus of the thalamus
ASSRs: Auditory steady-state responses
BF: Basal forebrain
CFC: Cross-frequency coupling
CSTC: Cortico-striato-thalamo-cortical
DBS: Deep brain stimulation
DMN: Default mode network
E/I: Excitatory-inhibitory
EEG: Electroencephalogram
ERP: Event-related potential
FGF: Fibroblast growth factors
GABA: γ-aminobutyric acid
GBO: γ band oscillations
GENUS: γ entrainment using sensory stimulation
GPT-4: Generative pre-trained transformer-4
HFO: High-frequency oscillations
HMM: Hidden markov model
ICA: Independent component analysis
ING: Interneuron network γ
IPSCs: Inhibitory postsynaptic currents
LLaMa-2: Large language model meta AI-2
LLMs: Large language models
MEG: Magnetoencephalography
mGluRs: Metabotropic glutamate receptors
NFB: Neurofeedback
OCD: Obsessive compulsive disorder
PAC: Phase amplitude coupling
PD: Parkinson’s disease
PING: Pyramidal-interneuron network γ
PLV: Phase-locking value
PTSD: Post-traumatic stress disorder
PV^+^: Parvalbumin-expressing
RSS: Rhythmic sensory stimulation
rTMS: Repetitive transcranial magnetic stimulation
SEEG: Stereo-electroencephalography
SOZ: Seizure onset zones
SQUID: Superconducting quantum interference device
SST^+^: Somatostatin-expressing
STN: Subthalamic nucleus
SWC: Spike-wave complex
tACS: Transcranial alternating current stimulation
TMS: Transcranial magnetic stimulation
VIP: Vasoactive intestinal peptide
VNS: Vagus nerve stimulation
